# Adaptive Responses of Thyroid Hormones, Insulin, and Glucose during Pregnancy and Lactation in Dairy Cows

**DOI:** 10.3390/ani12111395

**Published:** 2022-05-28

**Authors:** Esterina Fazio, Arianna Bionda, Vincenzo Chiofalo, Paola Crepaldi, Vincenzo Lopreiato, Pietro Medica, Luigi Liotta

**Affiliations:** 1 Department of Veterinary Sciences, Università di Messina, Viale Palatucci, 13, 98168 Messina, Italy; esterina.fazio@unime.it (E.F.); ariannabionda95@gmail.com (A.B.); vincenzo.chiofalo@unime.it (V.C.); pietro.medica@unime.it (P.M.); luigi.liotta@unime.it (L.L.); 2 Department of Agricultural and Environmental Sciences, Università di Milano, Via Celoria, 2, 20133 Milan, Italy; paola.crepaldi@unimi.it

**Keywords:** thyroid hormones, insulin, glucose, dairy cows

## Abstract

**Simple Summary:**

The effects of different stages of pregnancy and lactation on thyroid, insulin, and glucose responses in 30 dairy cows from different breeds were studied. Thyroxine (T_4_) and insulin were higher at the end of pregnancy than at >60–120 d or in nonpregnant cows. Along the lactation phase, T_4_ initially decreased, reaching the lowest values at >60–120 d, and then increased until the end of lactation. Glucose showed the highest concentrations during the first 120 days of lactation, and the lowest values at the end. The monitoring of thyroid hormones, insulin, and glucose changes represents an important tool to evaluate the anabolic and/or catabolic adaptation in response to functional periods in dairy cows, which can potentially predispose the cows to an excessive negative energy balance and related metabolic issues.

**Abstract:**

The study examined the effects of different stages of pregnancy and lactation on thyroid, insulin, and glucose responses in dairy cows. In the present study, 30 dairy cows (10 Holstein, 10 Simmental, and 10 Brown) at 30 ± 20 d of lactation were randomly selected and blood samples were collected once every 60 d for one year to measure circulating thyroid stimulating hormone (TSH), total and free triiodothyronines (T_3_, fT_3_) and thyroxines (T_4_, fT_4_), insulin, and glucose. Pregnant cows showed higher T_4_ (*p* = 0.010) and insulin (*p* = 0.046) concentrations at >180 d than at >60–120 d of pregnancy and in nonpregnant cows. Along the lactation phase, circulating T_4_ concentrations showed a biphasic trend, decreasing from 0–60 d to >60–120 d phase, which showed the lowest values, and then increasing until the end of lactation (>300 d) (*p* = 0.016). Glucose showed the highest concentrations at the start of lactation (0–120 days) and the lowest values at the end (*p* = 0.008). The monitoring of thyroid hormones, insulin, and glucose changes represents an important tool to evaluate the anabolic and/or catabolic adaptation in response to functional periods in dairy cows, which can potentially predispose the cows to an excessive negative energy balance and related metabolic issues.

## 1. Introduction

In the dairy cow, both pregnancy and lactation are characterized by consistent changes in the sensitivity and responses of tissues to the hormones involved in homeostasis, such as thyroid hormones (THs) and insulin [1].

Thyroid activity influences the reproductive axis functionality, promoting hormonal changes in the different reproductive periods [2,3,4]. There are several lines of evidence that both thyroglobulin and TSH receptors are present in bovine luteal cells [5] and that, in the presence of FSH and insulin, triiodothyronine (T_3_) has the ability to enhance aromatase activity in cultured bovine granulose cells [6]. Other in vitro studies confirmed that T_3_ and thyroxine (T_4_) directly promote steroidogenesis in follicular and luteal cells of different species, including cattle [6,7]. The concentrations of THs are particularly affected by the metabolic and nutritional processes of advanced pregnancy, and therefore are subjected to significant variations during the reproduction cycle. Hence, TH concentrations are high in dry cows and then significantly decrease in the peripartum period, especially in early lactation, when high milk production leads to an intense mobilization of the body reserves [8,9,10,11]. Indeed, growth hormone increases during the transition period, whereas insulin, THs, and insulin-like growth factor decrease [12]. A positive correlation between circulating THs and energy balance is well-known in cattle and many other species [13,14,15]. Thyroid hormones influence several metabolic pathways, including carbohydrate, protein, and lipid metabolism, thus increasing basal energy expenditure [16]. Thyroid activity is known to change with the stage of lactation [2]: in the first third of lactation, which is characterized by a negative energy balance, dairy cows show low T_3_ and T_4_, and an increase of fT_3_ concentrations [17,18,19], and the concentrations of T_3_ and T_4_ correlate negatively with milk yield [3].

The homeostatic control of the metabolism varies markedly throughout lactation as well [20]. Since the peak milk production, which occurs at about 8 to 10 weeks postpartum, precedes the maximum energy intake, the endocrine system needs to change accordingly: thus, the glucose and lipid metabolism are regulated in order to ensure the homeorhetic nutrient partitioning towards the mammary gland despite a catabolic state [21]. This selectivity in directing nutrients coincides with the reduced responsiveness and sensitivity of extrahepatic tissues to insulin, i.e., insulin resistance is thought to be markedly involved in developing ketosis and hepatic lipidosis [22].

The hypothesis of this study is that the adaptive contribution to metabolic changes along gestation and lactation in dairy cows could be mediated by changes of hormones with a pivotal role on the homeostatic control of the metabolism, such as THs and insulin. Therefore, we investigated circulating thyrotropic hormone (TSH), total (T_3_ and T_4_) and free (fT_3_ and fT_4_) thyroid hormones, insulin, and glucose responses in healthy Holstein, Simmental, and Brown dairy cows, according to different lactation and pregnancy periods.

## 2. Materials and Methods

### 2.1. Animals and Breeding

The experimental protocol was approved by the Ethical Committee of the Department of Veterinary Science, University of Messina, Italy (code 041/2020). The research complied with guidelines of Good Clinical Practices (EMEA, 2000) and the Italian and European regulations on animal welfare (Directive 2010/63/EU).

The present study included 30 multiparous dairy cows (10 Holstein, 10 Simmental, and 10 Brown), randomly selected from a large group of 100 animals, bred on the same commercial farm located in Ragusa (36°53′47″ N, 14°42′24.8″ E, 500 mt above sea level) (Italy), homogeneous for age (Holstein: 4.2 ± 1.5 years; Simmental: 3.9 ± 1.2 years; and Brown: 5.2 ± 1.8 years), body condition score (Holstein: 2.5 ± 0.3; Simmental: 3.4 ± 0.3; and Brown: 2.9 ± 0.3 at the time of the first sampling), lactation stage (30 ± 20 d), and average milk production (Holstein: 28.8 ± 3.3 kg/head/day; Simmental: 26.5 ± 2.8 kg/head/day; and Brown: 27.3 ± 3.1 kg/head/day) under the traditional semi-intensive farming system.

The nonpregnant phase was defined as the time interval between parturition and conception (on average 112 ± 80 d in lactation). All the included cows were inseminated and became pregnant at about 110–170 d of lactation.

All animals were considered healthy on the bases of: (i) normal cyclicity during the previous breeding seasons, (ii) absence of reproductive pathologies, and (iii) absence of inflammatory and infectious processes that occurred and were treated with antibiotics or anti-inflammatory agents within a month before the start of blood sampling and along the whole experimental period.

Animals were raised according to an approved UE disciplinary method called “QS Sicilia”, which allows for the inclusion of olive cake in feed for dairy cows up to 10% of the diet, as a strategy for the recovery of agro-industrial byproducts. For this reason, animals were fed with the same diet composed of ad libitum meadow hay and an average of 10 kg/head/day of concentrate integrated with 8% of dried and pitted olive cake (DM 95.6; CP 10.4; EE 15.9; NDF 49.4; ADF 39.4; ADL 23.1; ash 3.7; starch 1.5% as feed); the concentrate (5 kg/head/meal) was administered at 7:00 a.m. and 2:00 p.m. every day. Pasture was available in spring and autumn (for a minimum of 6 h during daylight, from 8:00 a.m. to 2:00 p.m.), but not in summer.

The animals had free access to water, both indoors and outdoors. The indoor housing was a free-stall barn equipped with automatic system fans and sprinklers that were activated during the hot season. Table S1 reports the mean temperature (T) and relative humidity (RH) recorded daily in Ragusa and the related temperature humidity index (THI), measured using the formula THI=(1.8×T+32)−[(0.55−0.55×RH)×(1.8×T−26.8)] [23].

### 2.2. Samples

All the animals were evaluated once every 60 d from February 2021 to February 2022. At the time of the first sampling, all the cows were at 30 ± 20 d of lactation. Blood sampling was performed at the same hour (7 a.m., before delivering concentrate, and it was always completed within an hour) through venipuncture from the jugular into 10 mL tubes containing clot activator and separating gel (Terumo Corporation, Tokyo, Japan). Blood samples were centrifuged for 10 min at 2000× *g*; the supernatant serum was collected and stored at −20 °C until analyses.

Serum TSH, T_3_, fT_3_, T_4_, fT_4_, insulin, and glucose concentrations were assessed using a human homologous solid-phase, two-site chemiluminescent immunometric assay (Immulite^®^ 2000, Siemens Medical Solutions, Diagnostics, Erlangen, Germany), according to the manufacturer’s instructions. All assays were validated for linearity using cows’ serum prior to use. The intra- and inter-assay coefficients of variation (CVs) were the following: for TSH, 5.5% and 9.5% at TSH concentrations of 0.2 and 2.35 ng/mL; for T_3_, 12% and 5.5% at T_3_ concentrations of 73 ng/dL and 171 ng/dL; for fT_3_, 9.1% and 5.4% at fT_3_ concentrations of 3.2 pg/dL and 13 pg/dL; for T_4_, 11.1% and 5.6% at T_4_ concentrations of 1.8 µg/dL and 16 µg/dL; and for fT_4_, 3.0% and 10.2% at fT_4_ concentrations of 4.82 ng/dL and 0.51 ng/dL, 1.56% and 4.07% at insulin concentrations of 16.54 and 45.804 µIU/mL. The sensitivity of the assay was 0.01 ng/mL for TSH, 19 ng/dL for T_3_, 1.0 pg/mL for fT_3_, 0.3 µg/dL for T_4_, 0.11 ng/dL for fT_4_, and 0.5 µIU/mL for insulin concentrations. Serum glucose concentrations were assessed by automated spectrophotometry (BT 3500, Biotecnica Instruments S.p.a., Roma, Italy) using the colorimetric enzymatic method GOD-POD.

### 2.3. Statistical Analyses

The software used for statistical analysis of the data was JMP^®^, Version 16 (SAS Institute Inc., Cary, NC, USA). Appropriate descriptive statistics were generated for all the analyzed variables.

Prior to analyses, data were subject to normality and homoscedasticity by Kolmogorov–Smirnov or Levene’s test. Logarithmic transformations were applied where necessary. ANOVA and post hoc Tukey–Kramer tests were used to identify significant (*p* < 0.05) differences among the different 60-day phases of lactation and pregnancy. The correlation between all the variables was expressed by Pearson’s correlation coefficient (r).

## 3. Results

### 3.1. Pregnant and Nonpregnant Cows

**Figure 1:**
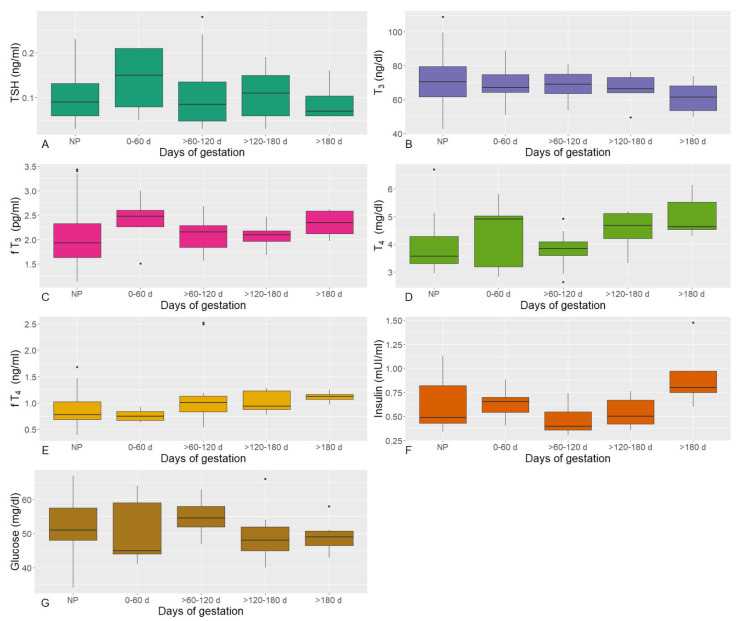
Boxplots of circulating thyroid-stimulating hormone (TSH, (**A**)), total triiodothyronine (T_3_, (**B**)), free triiodothyronine (fT_3_, (**C**)), total thyroxine (T_4_, (**D**)), free thyroxine (fT_4_, (**E**)), insulin (**F**), and glucose (**G**) in pregnant and nonpregnant (NP) cows.

Figure 1 and Figure S1 and Table S2 represent the circulating thyroid-stimulating hormone (TSH), total and free triiodothyronine (T_3_, fT_3_) and thyroxine (T_4_, fT_4_) concentrations, and insulin pattern measured in nonpregnant cows and along pregnancy. No significant differences were observed among Holstein, Brown, and Simmental cows in any of the pregnancy phases; therefore, they were considered as a single group.

Circulating TSH concentrations of cows showed a constant trend from 0–60 d of pregnancy to >120–180 d, with the lowest values at >180 d and in nonpregnant cows (Figure 1A). In pregnant cows, TSH ranged from 0.03 to 0.28 (mean ± standard deviation: 0.11 ± 0.067) ng/mL, whereas in nonpregnant ones, TSH ranged from 0.03 to 0.23 (0.09 ± 0.050) ng/mL.

Circulating T_3_ concentrations showed a constant trend from 0–60 d of pregnancy to >120–180 d, with the lowest values at >180 d; the highest values were observed in nonpregnant cows (Figure 1B). Pregnant cows showed concentrations of 49.5–88.9 (67.2 ± 9.27) ng/dL, whereas T_3_ was at 42.5–109.0 (71.2 ± 15.65) ng/dL in nonpregnant animals.

Circulating fT_3_ concentrations showed the highest values at 0–60 and at >180 d, with the lowest values at both >60–120 d and in nonpregnant cows (Figure 1C). Serum concentrations of fT_3_ measured during pregnancy ranged from 1.51 to 3.00 (2.17 ± 0.354) pg/mL; conversely, they ranged from 1.13 to 3.43 (2.08 ± 0.621) pg/mL in nonpregnant cows.

Circulating T_4_ concentrations showed higher values at ≥180 d (5.00 ± 0.770 mg/dL) and the lowest ones at >60–120 d (3.79 ± 0.626 mg/dL) and in nonpregnant cows (3.88 ± 0.835 mg/dL, ranging from 2.95 to 9.70 mg/dL) (*p* = 0.010) (Figure 1D). Considering the whole pregnancy, T_4_ ranged from 2.63 to 6.13 (4.28 ± 0.872) mg/dL.

Circulating fT_4_ tended to increase during pregnancy (*p* = 0.044); the concentrations measured in nonpregnant cows (0.86 ± 0.306, range: 0.39–1.68 ng/mL) were as low as the 0–60 d period (0.76 ± 0.131), but were more variable (Figure 1E). Overall, pregnant cows presented fT4 serum concentration from 0.53 to 2.52 (1.07 ± 0.427) ng/mL.

Circulating insulin concentrations were similar in nonpregnant cows (0.62 ± 0.258, ranging from 0.34 to 1.13 mUI/mL) and during the first two months of pregnancy (0–60 d, 0.64 ± 0.178 mUI/mL), with a decrease reaching the lowest values at >60–120 d (0.47 ± 0.150 mUI/mL), and then an increase peaking at >180 d of pregnancy (0.92 ± 0.382 mUI/mL) (*p* = 0.046) (Figure 1F). Considering all the pregnancy phases, insulin measurements ranged from 0.31 to 1.48 (0.59 ± 0.251) mUI/mL.

Circulating glucose concentrations showed the highest values at >60–120 d, with the lowest values at both >120–180 d and >180 d, with a trend that was opposite to the insulin (Figure 1G). Glucose measurement in pregnant cows ranged from 40 to 60 (52 ± 6.9) mg/dL, whereas in nonpregnant ones, it ranged from 34 to 67 (52 ± 7.7) mg/dL.

In pregnant cows, TSH significantly correlated with T_4_ (r = 0.39; *p* = 0.028), and a significant correlation was also found between T_4_ and T_3_ (r = −0.35; *p* = 0.041). Glucose was significantly correlated with T_4_ (r = −0.61; *p* < 0.001) and T_3_ (r = 0.46; *p* = 0.006).

Nonpregnant cows showed significant correlations between T_3_ and both fT_3_ (r = 0.54; *p* = 0.002) and glucose (r = 0.49; *p* = 0.005) and also between fT_3_ and fT_4_ (r = 0.63; *p* < 0.001).

### 3.2. Lactating Phases

**Figure 2:**
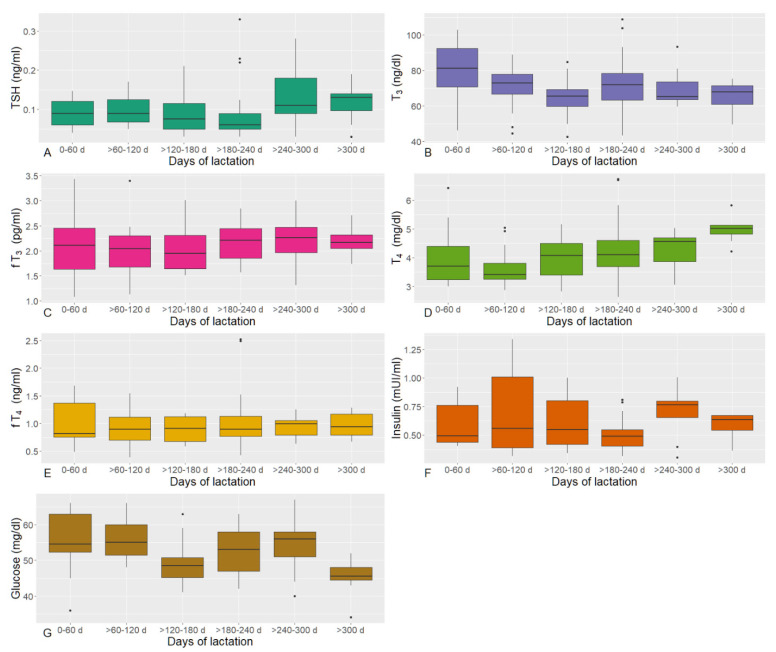
Boxplots of circulating thyroid-stimulating hormone (TSH, (**A**)), total triiodothyronine (T_3_, (**B**)), free triiodothyronine (fT_3_, (**C**)), total thyroxine (T_4_, (**D**)), free thyroxine (fT_4_, (**E**)), insulin (**F**), and glucose (**G**) in lactating cows.

Figure 2 and Figure S2 and Table S3 show the circulating thyroid-stimulating hormone (TSH), total and free triiodothyronine (T_3_, fT_3_) and thyroxine (T_4_, fT_4_) concentrations, insulin, and glucose pattern measured along the lactation phase. No significant differences were observed among Holstein, Brown, and Simmental cows in any of the lactation phases; therefore, they were considered as a single group.

Circulating TSH concentrations of dairy cows showed a constant trend from 0–60 d to >180–240 d of lactation, with a progressive increase from >240–300 to >300 d (Figure 2A). During the whole lactation phase, they ranged from 0.03 to 0.33 ng/mL, with a mean ± standard deviation of 0.10 ± 0.060 ng/mL.

Circulating T_3_ concentrations, which ranged from 42.5 to 109.0 (70.5 ± 13.74) ng/dL, were higher at the beginning of lactation, and then decreased; the lowest values were observed at >120–180 and >300 d of lactation (Figure 2B).

Circulating fT_3_ concentrations were almost constant throughout the lactation phase, ranging from 1.08 to 3.43 (2.13 ± 0.481) pg/mL, with slightly higher values at >240–300 d (Figure 2C).

Circulating T_4_ concentrations showed a biphasic trend, decreasing from 0–60 d (4.00 ± 1.052 mg/dL) to 60–120 d phase (3.65 ± 0.656 mg/dL), which showed the lowest values, and then increasing until the end of lactation (>300 d, 4.97 ± 0.466 mg/dL) (*p* = 0.017) (Figure 2D). Overall, T_4_ ranged from 2.63 to 6.72 (4.14 ± 0.909) mg/dL.

Circulating fT_4_ concentrations did not show evident changes throughout lactation, going from 0.39 to 2.52 (0.97 ± 0.371) ng/mL (Figure 2E).

Circulating insulin concentrations showed a variable but superimposed trend, with the highest values at >240–300 d of lactation and the lowest values at >180–240 d (Figure 2F); in particular, the minimum measured concentration was of 0.31 mUI/mL and the maximum was of 1.34 mUI/mL (0.60 ± 0.232 mUI/mL).

Lastly, glucose showed the highest concentrations at the start of lactation (0–60 d, 56 ± 9.3 mg/dL) until >60–120 days (56 ± 6.0 mg/dL), and the lowest values at the end (45 ± 5.3 mg/dL) (*p* = 0.008). Considering the whole lactation phase, circulating glucose ranged from 34 to 67 (53 ± 7.6) mg/dL.

Lactating dairy cows showed significant correlations between TSH and T_4_ (r = 0.24; *p* = 0.022), T_3_ and fT_3_ (r = 0.47; *p* < 0.001), T_4_ and fT_4_ (r = 0.26; *p* = 0.017), T_3_ and glucose (r = 0.56; *p* < 0.001), and T_4_ and glucose (r = −0.34; *p* = 0.002).

## 4. Discussion

The results obtained from the dairy cows included in the present study for serum TSH, T_3_, fT_3_, T_4_, fT_4_, and insulin concentrations were comparable to those found for this species by different authors [2,5,24,25,26,27]. The possible influence of circadian hormonal rhythms was limited by always performing the blood sampling at the same time for all the cows enrolled in the study.

The significant differences of T_4_ concentrations in pregnant cows, with higher concentrations at the end of pregnancy (>180 days) compared to nonpregnant cows, confirm the changes of THs observed during the advanced pregnancy, previously reported by different authors [8,9,10,11]. This result is in line with knowledge that approximately 75% of calf fetus growth, especially of adipose and muscle tissue [28], occurs during the last two months of pregnancy [29], with a related increase of nutrient requirements; for this reason, it is possible to presume that maternal THs can significantly affect calf growth during the last trimester of pregnancy. Moreover, it is well-known that ruminants’ fetus synthetizes endogenous THs during the second half of pregnancy [30,31,32,33]; thus, it is presumable that the higher T_4_ values at >180 days of pregnancy in dairy cows could also be due to the start in the calf’s thyroid activity, contributing to the maternal total iodothyronines homeostasis. Hence, as would be expected, the lowest values of T_4_ concentrations were recorded at >60–120 days of pregnancy. In addition, the significant negative correlation between T_4_ and T_3_ corroborated this hypothesis, with a presumably decreased secretion rate of T_3_ or its peripheral inactivation, or a decreased conversion of T_4_ to T_3_, according to the advanced pregnancy and dynamic energy expenditure. There is little evidence that, in ruminants, maternal THs cross the placenta into fetal circulation, at least in the second half of pregnancy [34,35], but the recent study of Steinhauser et al. [36] showed that several TH transporters are located in the ruminants’ placenta and that placental deiodinases regulate their availability to placental and fetal cells [36]. In this sense, our data would confirm the active metabolic role of THs in this species also, as previously recorded in pregnant goats [37], mares [38,39], and donkeys [40].

The prevalence of T_4_ synthesis along the pregnancy period was confirmed by the existence of a significant positive correlation between TSH and T_4_. The increase in the circulating T_4_ values detected in this study may have depended both on an increase in the thyrotropic activity of TSH and on a reduction in peripheral deiodinase activity, it being regulated by TSH [41]. These results corroborate the peculiar role of T_4_ secretion in metabolic adaptation to pregnancy and energy balance in dairy cows [42]. Moreover, the shift in substrate utilization, from fat to glucose, might contribute to reducing endogenous heat production and might at the same time preserve hepatic gluconeogenesis for fetal growth in late pregnancy and lactogenesis after calving [43]. It seems likely that the TH system and TH-mediated signaling play a pivotal role in the control of substrate utilization and thus also of cows’ body temperature.

Available evidence suggests that fT_4_ is the major secretory product of the thyrocyte, whereas circulating fT_3_ mainly comes from 5′deiodinase activity in peripheral tissues [41]. Given these observations, it is likely that the constant trend of fT_3_ and fT_4_ found in cows along different periods of pregnancy is due either to homeostatic activity of peripheral deiodinases approaching the pregnancy, or to a constant but dynamic secretory synthesis, respectively.

Likewise, the evidence of the highest T_4_ and insulin values recorded at >180 d of pregnancy is in line with their excitometabolic and anabolic roles, respectively; these effects are particularly intriguing because it is possible that the thyroid response to late pregnancy, meant to regulate energetic homeostasis, integrates hormonal signals that initiate energy conservation.

On the other hand, in nonpregnant dairy cows, the existence of significant and positive correlations between both T_3_:fT_3,_ and T_4_:fT_4_ showed the involvement of total and free THs in basal energy expenditure obtained activating the carbohydrate, protein, and lipid metabolism. Hence, the temporal and reversible shift of energy metabolism in a catabolic or anabolic direction is characterized by these wide observed correlations.

The significantly lowest T_4_ values observed at >60–120 d of lactation confirmed the decrease of THs during the first third of lactation [8,9,10,11,44], when the maximum energy intake occurs and the reserves are mobilized and used for high milk production. These last authors observed that circulating THs were lower at the beginning of lactation, then increased along the milk yield, and decreased again during the dry period. This decrease in T_4_ concentrations could favor the partitioning of nutrients between mammary and non-mammary tissue [42], decreasing the problems caused by nutrient deficiencies in body tissue occurring in the first phases of lactation. It must be noted that during lactation, the water metabolism to mammary gland through the vascular system is physiologically increased, thus causing possible hemodilution of the THs, as previously observed in lactating ewes for many hematological and biochemical substances [45].

On the other hand, the highest T_4_ values observed at >300 d, at the end of lactation, confirmed the negative correlation of THs with the milk yield.

With respect to the absence of significant differences in T_3_ and free iodothyronines along the milk yield, it is plausible that there was a large amount of individual variation in the animals along the analyzed physiological periods. Nevertheless, the significant positive correlations between T_3_:fT_3_ and T_4_:fT_4_ values observed in lactating phases are consistent with previous data observed in growing foals and pregnant goats, reporting that changes of total iodothyronine concentrations often follow those of the free form [37,46].

The insulin trend was variable but was overall constant during early and mid-lactation (0–240 d), followed by an increase in the last phase of lactation (>240–300). Circumstantial evidence suggests that the significant changes of insulin in early and mid-pregnancy may also reflect those of nutrient availability and catabolic or anabolic utilization, respectively [47]; this mismatch may temporarily alter the metabolic requirements to cope with the dynamic pregnancy phase. In addition, the highest concentrations of glucose at the start of lactation until >60–120 d, and the lowest values at the end, reflect the changes observed for insulin. This confirms that glucose is the most important substrate for milk production [48] and that insulin is a key regulator of glucose uptake by peripheral cells. Moreover, as previously recorded by Bossaert et al. (2008) [49], the lactating mammary gland’s insulin-independent glucose consumption leads to greater glucose clearance and supports nutrient fluxes to the mammary gland [50,51], making clear conclusions about peripheral insulin sensitivity difficult.

It has been shown in cattle that high environmental temperatures reduce circulating concentrations of T_4_ [52] and T_3_ [53,54], due to depression of the pituitary and thyroid components of the pituitary–thyroid–peripheral tissue axis, whereas normal capacity to generate T_3_ from T_4_ in the liver is preserved and remains unperturbed [55]. Li et al. (2006) [56] and Rhoads et al. (2013) [57] pointed out that proper insulin action is necessary to effectively mount a response to heat stress and minimize heat-induced damage. Contrariwise, according to Min et al. (2015) [58], no significant differences were observed in serum concentrations of insulin between heat stress and cooling. However, it is well-established that heat-stressed dairy cows experience a relevant reduction in feed intake, which prolongs the period of negative energy balance, and this may lead to decreased serum concentrations of insulin [59,60]. Therefore, heat stress may not directly affect the secretion of insulin. Based on our data, it is possible to exclude the influence of heat stress, since no marked differences in investigated hormones were observed between months with a THI lower or higher than 70. It is noteworthy, however, that in summer months, the THI was slightly higher than the breakpoint reported in dairy cows (the highest was 73.6 in August). Considering that cows during the summer were cooled in the barn and were not allowed to pasture, it can be hypothesized that cows might have experienced only mild heat stress.

Given the complexity of this adaptational system and the number of hormones involved, it is possible to presume that the dairy cows showed different strategies to cope with such stressors predominantly depending on the physiological stage. The prevailing high metabolic priority of milk production during the first months of lactation supported the nutrient fluxes to the mammary gland. The same is likely to occur during the early phase of fetal development, characterized by maximal placental growth, differentiation, and vascularization, but also during mid- and late pregnancy, when production-oriented tissues, such as muscle, develop. These metabolic priorities were corroborated by the existence of a significant negative correlation between T_4_ and glucose, and a positive one between T_3_ and glucose in both pregnant and lactating dairy cows, acknowledging the active metabolic role of T_3_. Thus, the shift of energy metabolism adapts differently to diminished or increased energy intake in both pregnant and lactating dairy cows, and a different THs’ sensitivity was involved in the regulation of catabolic or anabolic processes.

## 5. Conclusions

Unfortunately, a limited number of published results exist regarding the adaptive contribution of thyroid, insulin, and glucose responses along pregnancy and lactation phases in dairy cows.

Results obtained in the present study highlight the relative contribution of THs, insulin, and glucose to metabolic processes along the whole physiological pregnancy and to the milk yield of dairy cows, suggesting a significant involvement of T_4_ and insulin in pregnant dairy cows, and of T_4_ and glucose in lactating ones.

On the basis of the knowledge that THs have a significant effect on glucose metabolism and exert both insulin-agonistic and antagonistic actions in different organs [61], and that the concentrations of blood metabolites, such as glucose and insulin, may represent a tool for predicting postpartum disease risk and monitoring the herd [62,63,64], the persistent demands for knowledge about the relation between endocrine adaptation and reproductive/productive performances from both farmers and veterinarians underline that further research on this topic may be of great interest. The monitoring of thyroid hormones, insulin, and glucose changes could represent an important tool to evaluate the anabolic and/or catabolic adaptation in response to functional periods in dairy cows, which can potentially predispose the cows to an excessive negative energy balance and related metabolic issues. Further research is required to verify or dismiss the latter in order to definitively determine the optimal range of thyroid hormones, insulin, and glucose concentrations that is specific to different gestational and lactating phases of a greater number of animals.

## Data Availability

The data that support this study will be shared upon reasonable request to the corresponding author.

## Supplementary Materials

The following supporting information can be downloaded at: https://www.mdpi.com/article/10.3390/ani12111395/s1, Table S1: Mean ± SD environmental temperature and humidity recorded daily in Ragusa (https://www.wunderground.com/dashboard/pws/IRAGUSAD2, accessed on 31 March 2022), where the cows included in the present study were bred, and related temperature humidity index (THI). Table S2: Mean, standard deviation (SD), and range of measured serum concentrations of thyroid stimulating hormone (TSH), total (T_3_ and T_4_) and free (fT_3_ and fT_4_) thyroid hormones, insulin, and glucose in nonpregnant (NP) and pregnant dairy cows. Table S3: Mean, standard deviation (SD), and range of measured serum concentrations of thyroid-stimulating hormone (TSH), total (T_3_ and T_4_) and free (fT_3_ and fT_4_) thyroid hormones, insulin, and glucose in lactating dairy cows. Figure S1: Circulating thyroid stimulating hormone (TSH, A), total triiodothyronine (T3, B), free triiodothyronine (fT3, C), total thyroxine (T4, D), free thyroxine (fT4, E), insulin (F), and glucose (G) in pregnant and nonpregnant (NP) cows. Figure S2: Circulating thyroid stimulating hormone (TSH, A), total triiodothyronine (T3, B), free triiodothyronine (fT3, C), total thyroxine (T4, D), free thyroxine (fT4, E), insulin (F), and glucose (G) in lactating cows.
