# Supplementary material for: Adaptive Responses of Thyroid Hormones, Insulin, and Glucose during Pregnancy and Lactation in Dairy Cows
Source: Animals (Basel). 2022 May 28;12(11):1395. doi: 10.3390/ani12111395 (PMC9179583; doi:10.3390/ani12111395)
Supplement: Supplementary file 1 [file animals-12-01395-s001.zip › animals-1684290-SI.pdf]

Table S1. Mean ± SD environmental temperature and humidity recorded daily in Ragusa (https://www.wunderground.com/dashboard/pws/IRAGUSAD2), where the cows included in the present study were bred, and related temperature humidity index (THI). To limit heat stress, during the hot period, pasture was not available and the livestock housing was equipped with automatic system fans and freely accessible showers.

Period	Temperature (°C)	Humidity	THI	Lactation Stage	Pregnancy Stage
**February 2021**	12.9 ± 3.00	0.8 ± 0.06	55.7 ± 4.55	0 - 60 d	NP
**March 2021**	13.5 ± 1.41	0.4 ± 0.10	56.9 ± 1.62	0 - 60 d	NP
**April 2021**	16.3 ± 2.57	0.3 ± 0.06	60.1 ± 2.63	>60 - 120 d	NP
**May 2021**	20.3 ± 1.55	0.3 ± 0.06	64.3 ± 1.41	>60 - 120 d	NP
**June 2021**	25.0 ± 3.44	0.3 ± 0.07	69.3 ± 3.05	>120 - 180 d	0 - 60 d
**July 2021**	27.1 ± 1.65	0.3 ± 0.07	72.2 ± 1.40	>120 - 180 d	0 - 60 d
**August 2021**	28.1 ± 1.89	0.3 ± 0.07	73.6 ± 1.54	>180 - 240 d	>60 - 120 d
**September 2021**	25.5 ± 1.09	0.4 ± 0.07	71.3 ± 1.63	>180 - 240 d	>60 - 120 d
**October 2021**	20.3 ± 1.99	0.4 ± 0.11	65.2 ± 2.65	>240 - 300 d	>120 - 180 d
**November 2021**	18.5 ± 2.77	0.5 ± 0.13	63.4 ± 3.67	>240 - 300 d	>120 - 180 d
**December 2021**	13.1 ± 1.82	0.4 ± 0.12	56.5 ± 2.24	>300 d	>180 d
**January 2022**	11.7 ± 1.86	0.4 ± 0.11	54.8 ± 2.04	>300 d	>180 d
**February 2022**	12.9 ± 1.13	0.4 ± 0.15	56.3 ± 1.27		>180 d

Table S2. Mean, standard deviation (SD), and range of measured serum concentrations of thyroid stimulating hormone (TSH), total (T_3 and T_4) and free (fT_3 and fT_4) thyroid hormones, insulin, and glucose in nonpregnant (NP) and pregnant dairy cows. P-values refer to ANOVA test results comparing the different pregnancy phases; within each row, different superimposed letters indicate significant differences between phases, according to the post hoc Tukey–Kramer test.

Pregnancy Stage		NP	0 - 60 d	>60 - 120 d	>120 - 180 d	>180	p-Value
TSH (ng/ml)	Mean ± SD	0.09 ± 0.05	0.14 ± 0.08	0.11 ± 0.08	0.11 ± 0.06	0.09 ± 0.04	0.749
	Range	0.03 - 0.23	0.05 - 0.21	0.03 - 0.28	0.03 - 0.19	0.06 - 0.16	
T_3 (ng/dL)	Mean ± SD	71.2 ± 15.65	69.2 ± 14.01	69.3 ± 7.75	66.6 ± 8.00	61.3 ± 9.63	0.505
	Range	42.5 - 109.0	50.8 - 88.9	53.6 - 81.0	49.5 - 76.5	49.8 - 73.9	
fT_3 (pg/ml)	Mean ± SD	2.08 ± 0.62	2.37 ± 0.55	2.10 ± 0.34	2.08 ± 0.24	2.33 ± 0.28	0.647
	Range	1.13 - 3.43	1.51 - 3.00	1.57 - 2.68	1.68 - 2.47	1.97 - 2.62	
T_4 (mg/dl)	Mean ± SD	3.88^{B} ± 0.84	4.35^{AB} ± 1.28	3.79^{B} ± 0.63	4.51^{AB} ± 0.66	5.00^{A} ± 0.77	0.010
	Range	2.95 - 6.70	2.82 - 5.81	2.63 - 4.92	3.31 - 5.19	4.29 - 6.13	
fT_4 (ng/ml)	Mean ± SD	0.86^{A} ± 0.31	0.76^{A} ± 0.13	1.16^{A} ± 0.60	1.04^{A} ± 0.21	1.11^{A} ± 0.10	0.043
	Range	0.39 - 1.68	0.63 - 0.92	0.53 - 2.52	0.77 - 1.28	0.97 - 1.25	
Insulin (mUI/ml)	Mean ± SD	0.62^{AB} ± 0.26	0.64^{AB} ± 0.18	0.47^{B} ± 0.15	0.54^{AB} ± 0.17	0.92^{A} ± 0.38	0.046
	Range	0.34 - 1.13	0.41 - 0.88	0.31 - 0.74	0.36 - 0.76	0.60 - 1.48	
Glucose (mg/dl)	Mean ± SD	52 ± 7.8	51 ± 10.2	55 ± 5.2	49 ± 7.5	49 ± 5.1	0.330
	Range	34 - 67	41 - 64	47 - 63	40 - 66	43 - 58	

Table S3. Mean, standard deviation (SD), and range of measured serum concentrations of thyroid-stimulating hormone (TSH), total (T_3 and T_4) and free (fT_3 and fT_4) thyroid hormones, insulin, and glucose in lactating dairy cows. P-values refer to ANOVA test results comparing the different lactation phases; within each row, different superimposed letters indicate significant differences between phases, according to the post hoc Tukey–Kramer test.

Lactation Stage		0 - 60 d	>60 - 120 d	>120 - 180 d	>180 - 240 d	>240 - 300 d	>300 d	p-Value
TSH (ng/ml)	Mean ± SD	0.09 ± 0.036	0.10 ± 0.040	0.09 ± 0.053	0.09 ± 0.078	0.14 ± 0.071	0.12 ± 0.050	0.102
	Range	0.04 - 0.15	0.05 - 0.17	0.03 - 0.21	0.03 - 0.33	0.03 - 0.28	0.03 - 0.19	
T_3 (ng/dL)	Mean ± SD	79.3 ± 18.03	70.1 ± 11.95	65.3 ± 11.19	71.8 ± 15.62	69.6 ± 9.72	65.1 ± 9.71	0.132
	Range	46.1 - 103.0	44.5 - 88.9	42.5 - 84.9	43.3 - 109.0	59.5 - 93.4	49.5 - 75.2	
fT_3 (pg/ml)	Mean ± SD	2.17 ± 0.748	2.04 ± 0.531	2.05 ± 0.452	2.16 ± 0.384	2.22 ± 0.442	2.19 ± 0.289	0.893
	Range	1.08 - 3.43	1.13 - 3.40	1.51 - 3.01	1.57 - 2.84	1.32 - 3.00	1.74 - 2.71	
T_4 (mg/dl)	Mean ± SD	4.00^{AB} ± 1.052	3.65^{B} ± 0.656	3.96^{AB} ± 0.738	4.27^{AB} ± 1.146	4.35^{AB} ± 0.548	4.97^{A} ± 0.466	0.016
	Range	2.99 - 6.42	2.87 - 5.04	2.82 - 5.16	2.63 - 6.72	3.06 - 5.02	4.21 - 5.81	
fT_4 (ng/ml)	Mean ± SD	1.01 ± 0.408	0.90 ± 0.315	0.88 ± 0.225	1.04 ± 0.543	0.95 ± 0.205	0.97 ± 0.238	0.933
	Range	0.49 - 1.68	0.39 - 1.54	0.59 - 1.18	0.43 - 2.52	0.63 - 1.25	0.67 - 1.28	
Insulin (mUI/ml)	Mean ± SD	0.59 ± 0.186	0.69 ± 0.344	0.62 ± 0.256	0.50 ± 0.141	0.70 ± 0.221	0.58 ± 0.146	0.340
	Range	0.41 - 0.92	0.31 - 1.34	0.34 - 1.00	0.32 - 0.81	0.31 - 1.01	0.36 - 0.67	
Glucose (mg/dl)	Mean ± SD	56^{A} ± 9.3	56^{A} ± 6.0	50^{AB} ± 7.0	52^{AB} ± 6.7	54^{AB} ± 8.2	45^{B} ± 5.3	0.008
	Range	36 - 66	48 - 66	41 - 63	42 - 63	40 ± 67	34 - 52	

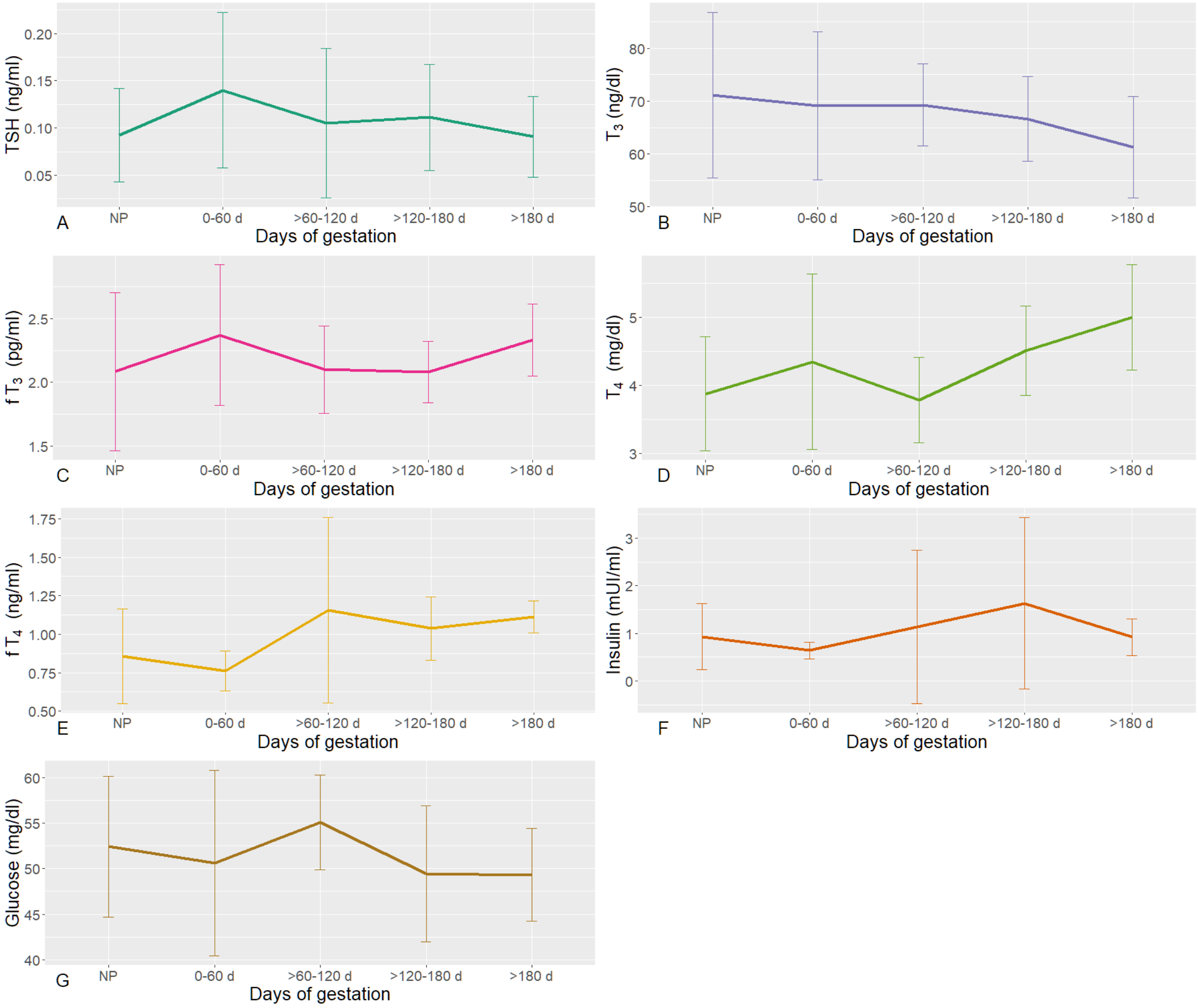

Figure S1. Circulating thyroid stimulating hormone (TSH, A), total triiodothyronine (T3, B), free triiodothyronine (fT3, C), total thyroxine (T4, D), free thyroxine (fT4, E), insulin (F), and glucose (G) in pregnant and nonpregnant (NP) cows.

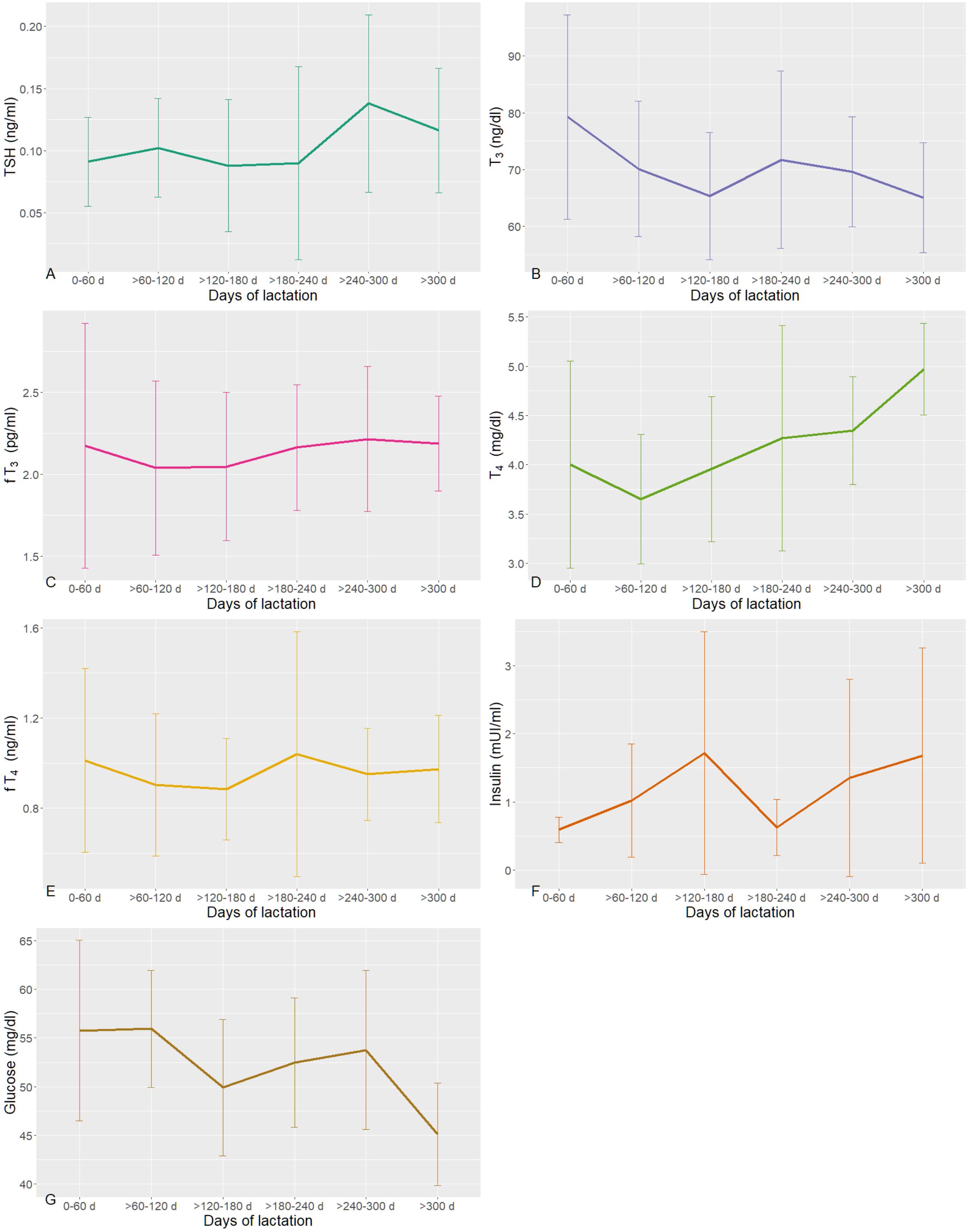

Figure S2. Circulating thyroid stimulating hormone (TSH, A), total triiodothyronine (T3, B), free triiodothyronine (fT3, C), total thyroxine (T4, D), free thyroxine (fT4, E), insulin (F), and glucose (G) in lactating cows.
